# Opportunities and challenges for the biodegradable magnesium alloys as next-generation biomaterials

**DOI:** 10.1093/rb/rbw003

**Published:** 2016-03-23

**Authors:** Wenjiang Ding

**Affiliations:** Shanghai Jiao Tong University, 800 Dongchuan Road, Shanghai 200240, China

**Keywords:** biodegradable material, magnesium alloys, uniform corrosion, orthopaedic implants, cardiovascular stents

## Abstract

In recent years, biodegradable magnesium alloys emerge as a new class of biomaterials for tissue engineering and medical devices. Deploying biodegradable magnesium-based materials not only avoids a second surgical intervention for implant removal but also circumvents the long-term foreign body effect of permanent implants. However, these materials are often subjected to an uncontrolled and fast degradation, acute toxic responses and rapid structural failure presumably due to a localized, too rapid corrosion process. The patented Mg–Nd–Zn–based alloys (JiaoDa BioMg [JDBM]) have been developed in Shanghai Jiao Tong University in recent years. The alloy series exhibit lower biodegradation rate and homogeneous nanophasic degradation patterns as compared with other biodegradable Mg alloys. The in vitro cytotoxicity tests using various types of cells indicate excellent biocompatibility of JDBM. Finally, bone implants using JDBM-1 alloy and cardiovascular stents using JDBM-2 alloy have been successfully fabricated and *in vivo* long-term assessment via implantation in animal model have been performed. The results confirmed the reduced degradation rate *in vivo*, excellent tissue compatibility and long-term structural and mechanical durability. Thus, this novel Mg-alloy series with highly uniform nanophasic biodegradation represent a major breakthrough in the field and a promising candidate for manufacturing the next generation biodegradable implants.

## Opportunities for the biodegradable Mg alloys as next-generation biomaterials

In the last few decades, non-degradable metals, namely titanium and titanium alloys and stainless steels, have been the most widely used biomaterials for orthopaedic implants. However, the major limitations of these currently applied metals lie in their undesirable mechanical properties, leading to the serious stress-shielding problem [1], and the non-degradability of these permanent materials, thus requiring a second surgery for implant removal, as well as the release of toxic ions through corrosion or wear process, such as titanium particles, which could even cause inflammatory osteolysis [2, 3]. Therefore, an urgent necessity for the development of next-generation implant biomaterials arises.

Recently, biodegradable metallic materials have gained growing interest and are intensively investigated, among which magnesium (Mg) and magnesium alloys emerge as a new class of promising candidate [4, 5]. Magnesium exhibits several key advantages with respect to biomedical applications, especially for load-bearing orthopaedic and cardiovascular implants:

i) Most importantly, it owns a natural ability to biodegrade due to corrosion within aqueous substances especially that contain chloride ions. The overall corrosion reaction that magnesium undergoes in aqueous solutions is given below:

(1) $$\text{Mg }+{\text{ 2H}}_{\text{2}}\text{O }\;\to \text{ Mg}{\left(\text{OH}\right)}_{\text{2}}+{\text{ H}}_{\text{2}}↑$$

(2) $$\text{Mg}{\left(\text{OH}\right)}_{\text{2}}\;\underset{\to }{{\text{Cl}}^{-}}\;{\text{Mg}}^{\text{2+}}\text{ + }{\text{2OH}}^{-}$$

Thus, it provides a theoretical basis for Mg and Mg alloys as temporary implants to support tissue regeneration and healing by material dissolution/degradation and concurrent implant resorption/replacement with the surrounding tissue.

**Figure 1.**
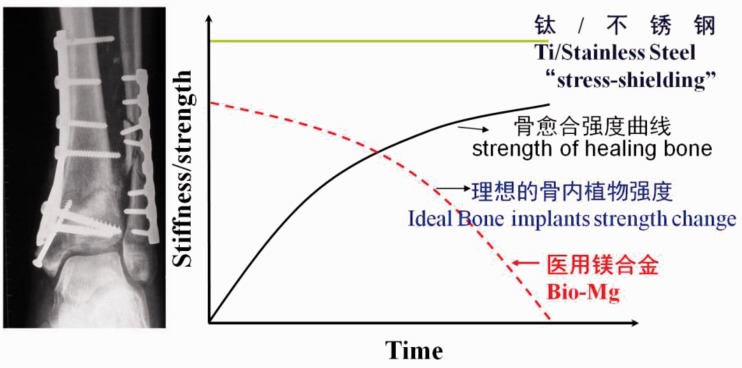
Mechanical property matching between new bones and implants

For orthopaedic internal fixation application, as the mechanical properties of healing bones gradually increase with the fracture recovery, correspondingly, ideal bone implants should possess a decreasing strength change adapted to the healing process. Deploying biodegradable magnesium and magnesium alloys thus offers a great opportunity to achieve synchronized strength change, whereas the mechanical properties of permanent implants (titanium and stainless steels) remain almost constant during the whole healing process, causing stress shielding problem (Fig. 1). Additionally, the biodegradability of magnesium also obviates the need for a second operation to remove implant once the healing process completes, reducing the cost and pain for patients.

**Figure 2.**
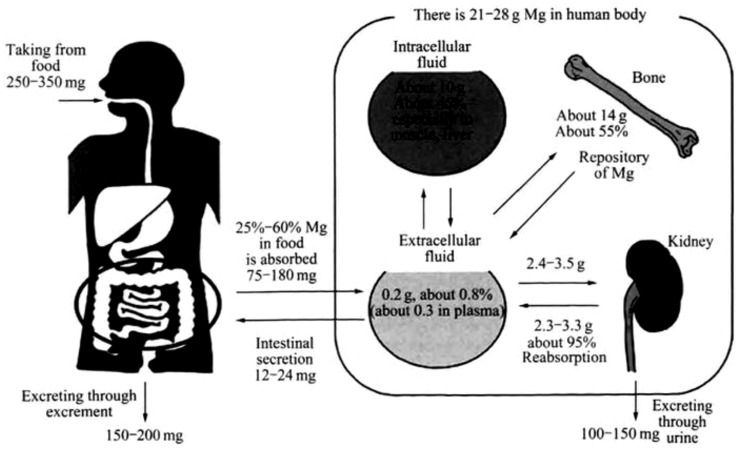
Distribution of dynamic absorption and excretion equilibrium of Mg in human body [8]

In the field of cardiovascular implants, stenting is the mainstay of surgical treatments to rescue the function of diseased coronary arteries [6]. The use of conventional permanent materials, including stainless steel, nickel–titanium alloys, tantalum and nitinol, due to the non-degradability of the materials, results in possible issues such as late restenosis (∼20%), chronic inflammation, mechanical blockages of the ostia of side branches, late development of malapposition leading to ectatic or aneurismal formation [7]. This pushes the development of fully reabsorbable stent as the future trend. Biodegradable Mg and Mg alloys can be used as a short-term structural support and allows for recovery of arteries without leaving behind foreign materials, offering the possibility of enhanced wound healing and reconstruction of vascular compliance while minimizing chronic inflammatory response and reducing the rate of late restenosis.

ii) It exhibits good biosafety and biocompatibility profile. Magnesium is an essential trace element in human body, namely the fourth most abundant mineral, and is necessary as a cofactor in more than 325 different enzymatic reactions, playing an important role in energy metabolism. It is required for the proper functioning of the heart, muscles, nerves, bones and kidneys. Recommended by the WHO, adults need an amount of 280–300 mg daily intake, while 250 mg for children and 80 mg for infants, respectively. Serum magnesium levels can be maintained normal due to the dynamic absorption and excretion equilibrium of magnesium in human body, through gastrointestinal absorption and renal excretion (Fig. 2) [8].

Additionally, the other degradation products H_2_ is also reported to be beneficial to health, as it has an antioxidant effect, identified as a selective scavenger of hydroxyl radical and peroxynitrite. The degradation products of magnesium can all be metabolized and absorbed by the body, and thus a solid foundation laid for Mg and Mg alloys as biodegradable implant materials regarding biosafety issues.

iii) It possesses superior mechanical properties required to meet loading requirements as for orthopaedic implants. The density, elastic modulus and compressive yield strength of magnesium are quite close to those of natural bones compared with titanium alloys or stainless steels (see Table 1) [9], and thus minimize the level of stress shielding. In the aspect of stent application, magnesium has similar mechanical strength to stainless steel and other stenting metallic materials to provide sufficient radial support, whereas biodegradable polymeric stenting materials (e.g. polylactic acid (PLA)) own much lower radial strength so that thicker stent struts are required. Thanks to these desirable physical and mechanical properties, Mg and Mg alloys are endowed with great potential as ideal temporary implant materials.

**Table 1.** Summary of physical and mechanical properties of various implant materials in comparison with natural bone ^[^10^]^

Characteristics	Cortical bone	Mg alloy	Ti alloy	316L stainless steel
**Density** (g/cm^3^)	1.8–2.1	1.74–2.1	4.4–4.5	7.9–8.1
**Elastic modulus** (GPa)	7–30	44–45	110–117	189–205
**Compressive yield strength** (MPa)	130–180	130–380	750––1200	170–310

Moreover, magnesium would not interfere with common radiological examinations for post-operative care, such as CT and MRI. These aforementioned desirable properties have led to worldwide attention on Mg-based biomaterials, known as the ‘revolutionizing metallic biomaterial’[10, 11], for tissue engineering and medical devices.

## Challenges for the bio-Mg clinical applications

Currently, there still exist several technical challenges impeding the clinical applications of biodegradable Mg and Mg alloys. First, pure magnesium and some magnesium alloys can corrode too quickly under physiological conditions, resulting in early loosening or disintegration of the implants before the tissue has sufficiently healed, as well as the rapid release of hydrogen gas in a short period of time in the corrosion process that is too fast to be dealt with by the host tissue. Secondly, Mg and Mg alloys are highly susceptible to localized and inhomogeneous degradation, leading to local stress concentration and decrease of mechanical strength, consequently subjecting the implants to unexpected fracture way before the anticipated lifetime. Last, the strength and ductility of Mg and Mg alloys are still required to be improved for specific applications so as to achieve optimal *in vivo* performance. Hence, to develop novel Bio-Mg alloys for medical applications is essential to the ultimate realization of clinical use.

Regarding principles of material design, the following key scientific issues need to be borne in mind:

- Biocompatibility and biosafety. Elements such as Al, which are not suitable for biomedical applications in consideration of toxicity, should be avoid for alloying.
- Compatible strength and ductility. As for orthopaedic implants, material is required to possess yield strength >200 MPa, elongation >10% and a degradation rate <0.5 mm/a in simulated fluids at 37°C, to ensure an effective lifetime of 90–180 days. While for cardiovascular stents, higher ductility and moderate strength is desirable, namely elongation >20% [12].
- Controllable degradation. Most reported Mg alloys are easily subjected to localized corrosion. Nevertheless, uniform and controllable degradation behaviours are crucial for accurate predictions of implant serving lifetime.

These three aspects are highly interrelated with respect to the design and engineering of Mg alloys. Thus, how to choose biocompatible alloying elements to achieve compatible mechanical properties, meanwhile to ensure controllable degradation through alloy composition design and microstructure design and control, has posed a great challenge in the development of novel Bio-Mg.

## Research progress in bone implants and cardiovascular stents *in situ*

### Development of novel JDBM Mg alloys

Recently, a new type of patented Mg–Nd–Zn-based Bio-Mg alloys (Jiaoda BioMg, denoted as JDBM) alloy has been developed in Shanghai Jiao Tong University [13–15], using first principal calculations and molecular dynamics simulation, combined with experimentation. In this Bio-Mg alloy series, neodymium was selected as the main alloying element, accompanied with the microalloyed Zn and Zr. Nd is one of light rare earth elements barely shown cytotoxicity, the addition of whom has already exhibited signiﬁcant strengthening effect in Mg-Nd binary alloys [16], and could greatly slow down galvanic corrosion of matrix. Zn is one of the abundant nutritionally essential elements in the human body, which enhances the ductility and deformability of Mg alloy. Zr is an effective grain-refining agent in Al-free magnesium alloys that contributes to strengthening, with verified biocompatibility in a magnesium alloy [17]. Other than the contribution of the above alloying elements, thermo-mechanical processing, namely hot extrusion, is employed to achieve grain refining, improving mechanical properties and generating residual compressive stresses in the subsurface of Mg alloys to reduce the corrosion rate [18, 19].

**Figure 3.**
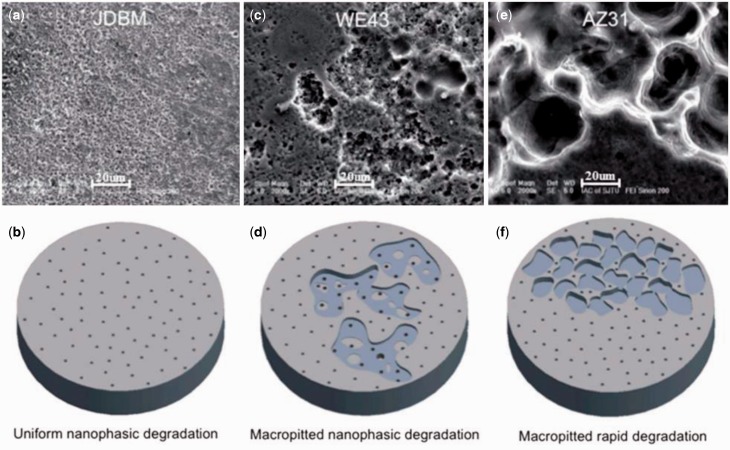
Surface morphology and the schematic diagram of the corresponding degradation mechanism of JDBM **(a and b)**, WE43 **(c and d)** and AZ31 **(e and f)** alloys. The surface of the new JDBM alloy upon exposure to artificial plasma displays a highly uniformarray of nanopits with typical size <500 nm. In contrast, both WE43 and AZ31 alloys show macroscopic pitting or delamination that dominates the degradation process and results in a fast degradation rate and ultimately structural failure [22]

**Figure 4.**
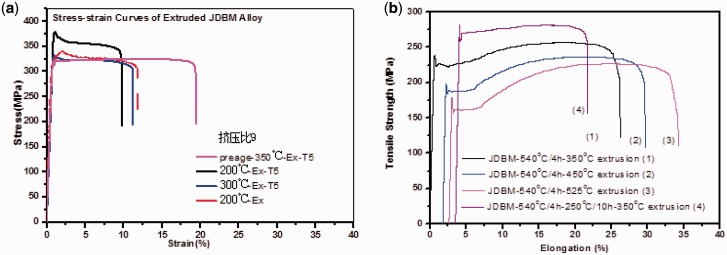
Mechanical properties of **(a)** JDBM-1 with high strength and moderate ductility; and **(b)** JDBM-2 with high ductility and moderate strength

**Figure 5.**
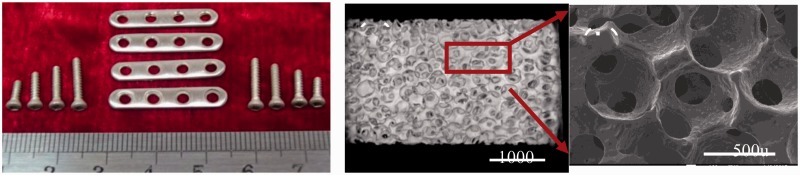
Various bone implants fabricated using JDBM-1 alloy, including bone plates, screws and porous bone tissue scaffold

**Figure 6.**
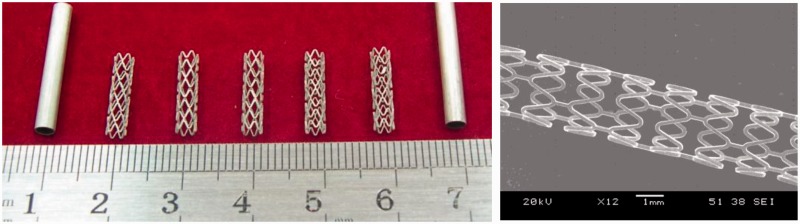
Cardiovascular stents fabricated using JDBM-2 alloys

As a result, our novel JDBM materials have been proven to be a type of biocompatible magnesium alloys of tunable mechanical properties for different specific applications, with a massive superiority in nanophasic uniform and slow degradation over many commercial available Mg alloys, such as AZ 31 and WE43 (Fig. 3) [13, 14]. So far, mainly two series of JDBM have been developed: (i) JDBM-1 with high strength and moderate ductility for orthopaedic implants. Tensile strength and yield strength = 320–380 MPa, and elongation =8–18%, as shown in Figure 4a. A variety of bone implants have been generated with JDBM-1 material, including bone plates, screws and even porous bone tissue scaffolds (Fig. 5); (ii) JDBM-2 with high ductility and moderate strength for cardiovascular stents (Fig. 6). Tensile strength and yield strength = 180–280 MPa, and elongation =20–32%, as shown in Figure 4b.

### JDBM-1 alloy for orthopaedic implants application

**Figure 7.**
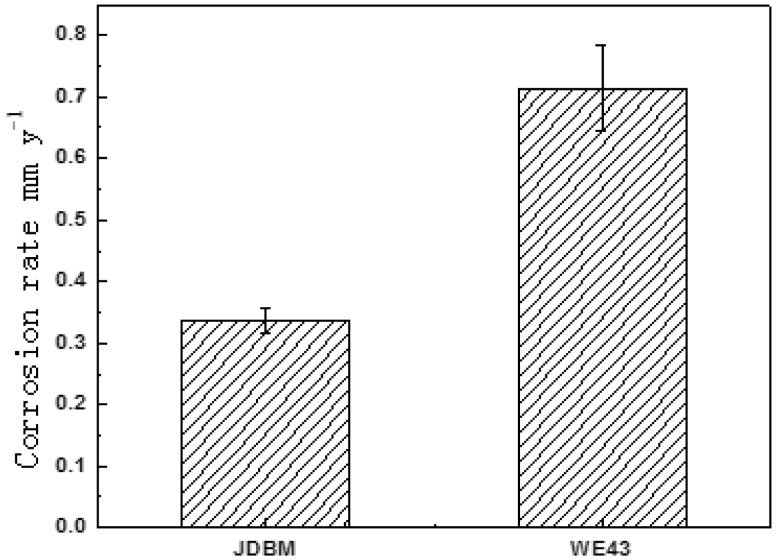
*In vitro* corrosion rate measured with immersion test showing a much lower rate of JDBM compared with that of WE43 [23]

**Figure 8.**
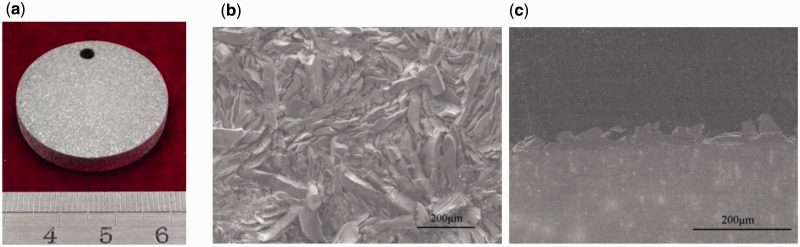
(a) Macroscopic picture of a brushite-coated JDBM sample, **(b)** scanning electron microscope (SEM) image of the brushite coating on JDBM substrate and **(c)** cross-section view of the brushite coating [24]

Previous studies have shown that JDBM-1 has much lower degradation rate, homogeneous corrosion property and slightly higher cytocompatibility, compared with clinical trial WE 43 (Fig. 7) [13]. Additionally, a bioactive Ca–P coating (brushite, CaHPO_4_·2H_2_O) with high bonding strength with substrate over 10 MPa was developed successfully on JDBM-1 alloy through chemical reaction deposition (Fig. 8, further improving the biocorrosion resistance and biocompatibility [20]. Both *in vitro* and *in vivo* results confirmed that brushite-coated JDBM-1 revealed adequate biosafety and biocompatibility and presented advantages in osteoconductivity and osteogenesis as bone repair substitutes [20, 21].

**Figure 9.**
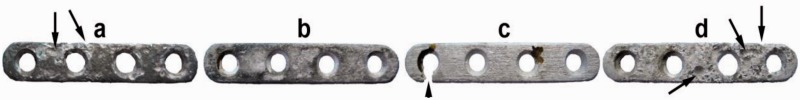
*In vivo* degradation morphology of bone plates fabricated with **(a)** JDBM, **(b)** JDBM covered with brushite coating, **(c)** AZ31 and **(d)** WE43, at 18 weeks post-implantation into rabbits

The *in vivo* experiments with bone plates in New Zealand rabbits up to 18 weeks showed effective biocorrosion resistance of JDBM-1 in the early stage post implantation. At 18 weeks, both JDBM and coated-JDBM plates revealed homogeneous corrosion profile and retained most part of the original strength (52 and 70%, respectively) whereas WE43 and AZ31 exhibited serious localized corrosion with much lower strength left (37%) (Fig. 9).

Currently, *in vivo* implantation in big animal model of goat has being carried out with JDBM-1 bone fixation screws, using commercially available PLA screws as control group. Preliminary results showed a good recovery of the wound by appearance after 40 days post implantation with JDBM-1 screws. CT results at 8 weeks post-implantation verified better biocompatibility and faster bone recovery with JDBM-1, along with hard tissue section evaluations further confirming higher new bone formation ability, compared with those with PLA screws. These promising results indicate that the JDBM-1 alloy possesses great potential for clinical application as orthopaedic implant materials.

### JDBM-2 alloy for cardiovascular stents application

Along with the development of JDBM-2 alloy with high ductility and moderate strength, mini-tube processing techniques and surface electropolishing conditions have also been established for the generation of prototype JDBM-2 cardiovascular stents.

**Figure 10.**
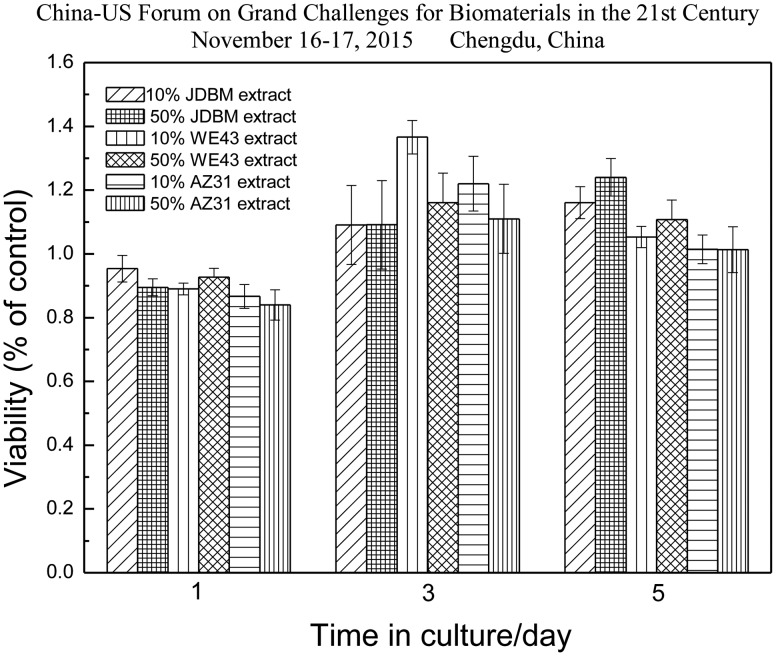
Statistical results of cytotoxicity assays using Human Umbilical Vein Endothelial Cells (HUVEC) incubated with JDBM, WE43, AZ31 extracts, respectively [25]

**Figure 11.**
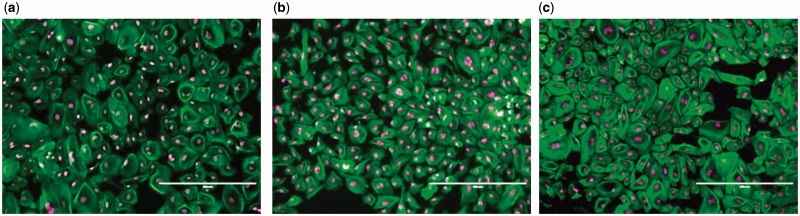
HUVEC morphology after incubated for 24 h with the extracts of (a) negative control, **(b)** JDBM-2 and **(c)** WE43 [25]

**Figure 12.**
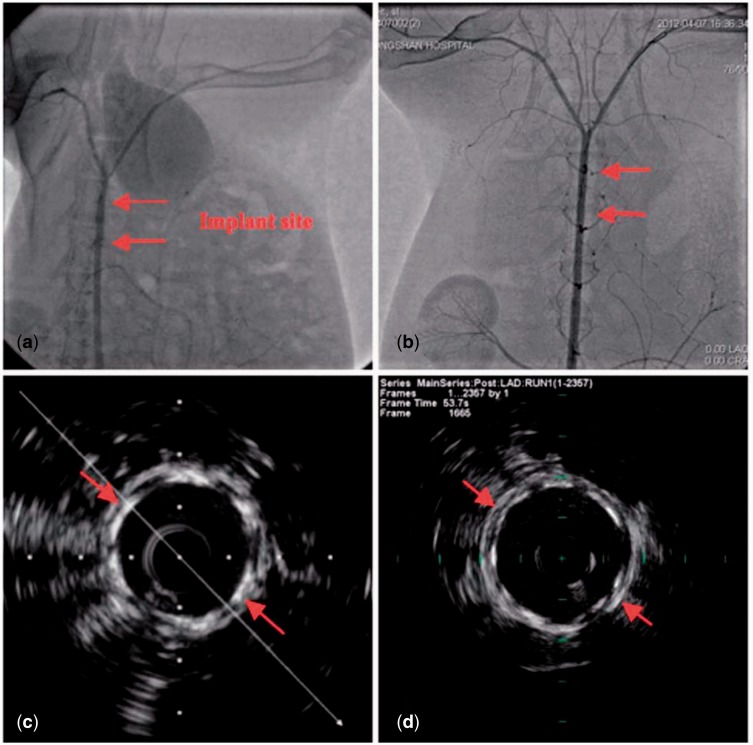
The as-implanted **(a and c)** and 16-week follow-up **(b and d)** angiographic and the corresponding longitudinal reconstruction Intravascular Ultrasound (IVUS) images of the abdominal aorta after JDBM stent implantation. Note the increased plaque volume and vessel size (arrow heads) at 16 weeks with the nearly complete absence of neointimal hyperplasia [26]

A systematic study on the *in vitro* biocompatibility test of JDBM-2 in comparison with two conventional degradable alloys (WE43 and AZ31) used for vascular stents verifies that JDBM-2 has a minimal negative effect on the HUVEC viability (Fig. 10), growth and proliferation and the JDBM-2 substrate offers a much more favourable surface for endothelial cell adhesion and spreading (Fig. 11) [14]. Furthermore, *in vivo* experiment with a rabbit model for long-term suggests that the JDBM-2 alloy stent has significantly improved mechanical durability and long-term biocompatibility with no sign of the development of occlusion and neointimal formation in the stent-supported vessel (Fig. 12) [14]. Preliminary results with big animal (pig) model further confirmed the superiority of JDBM-2 stents, showing good mechanical integrity and sufficient supporting function and no obvious degradation or displacement in the follows up of 2 weeks and 3 months with OCT examination, thus is a promising material for future biodegradable vascular stent applications.

## Concluding remarks

Biodegradable Mg-based material is a new research field of both great academic and marketing importance. Though it has shown good application prospect, the following three key scientific issues strength and ductility, controllable degradation and biosafety, are urgently required to be solved. In the middle of the difficulty lies opportunity!

A novel patented Mg-Nd-Zn-Zr alloy series, JDBM, with unique uniform and slow degradation characteristics, has been developed and researched in Shanghai Jiao Tong University, as a potential biodegradable implant material. Both *in vitro* and *in vivo* evaluations have verified the superiority of JDBM with respect to degradation rate and mode, biocompatibility and bioactivity, for orthopaedic implants and cardiovascular stents. Our research achievements are expected to be applied to clinic application in the near future of 3–5 years.
